# The effect of expertise on postural control in elite sport ju-jitsu athletes

**DOI:** 10.1186/s13102-022-00477-3

**Published:** 2022-05-13

**Authors:** Anna Akbaş, Wojciech Marszałek, Sławomir Drozd, Wojciech Czarny, Paweł Król, Krzysztof Warchoł, Kajetan J. Słomka, Marian Rzepko

**Affiliations:** 1 Department of Motor Human Behavior, Institute of Sport Sciences, Academy of Physical Education, 72a Mikołowska Str., 40-065 Katowice, Poland; 2 Institute of Physical Culture, University of Rzeszów, 16c Aleja Rejtana Str., 35-959 Rzeszów, Poland

**Keywords:** Rambling-trembling, Sample entropy, Ecological conditions, Equilibrium

## Abstract

**Background:**

Due to the high postural control demands of sport ju-jitsu, it is likely that long-term sport ju-jitsu training may induce sport-specific adaptations in postural control, especially in positions directly related to combat. The aim of the study was to assess the differences in postural control between elite sport ju-jitsu athletes and untrained controls in non-ecological and ecological conditions and to investigate the relative contribution of spinal and supraspinal mechanisms to postural control in expert athletes.

**Methods:**

The study was conducted on eleven male elite ju-jitsu athletes and ten non-athletes. The data was collected with the use of a force plate under two conditions: quiet standing and ju-jitsu combat stance. Apart from the standard analysis of the spatial–temporal parameters of center of foot pressure, non-linear measures were used, namely rambling-trembling and sample entropy. The non-parametric Mann–Whitney U test was used to compare both groups.

**Results:**

The main findings of the study showed that in quiet standing, elite ju-jitsu athletes and non-athletes had comparable postural control in both the anterior–posterior and mediolateral planes. In contrast, in the combat stance, elite athletes had lower values of postural sway and rambling component (range and rms) and higher values of sample entropy in comparison to the non-athletes (*p* < 0.05). No differences were found in the trembling component of sway between groups in the combat stance (*p* < 0.05).

**Conclusions:**

Smaller postural sway and its rambling component in sport ju-jitsu athletes may indicate the more precise control of center of foot pressure and improved ability in estimating its position. The sample entropy results confirmed that the displacement of center of foot pressure for sport ju-jitsu athletes was more irregular, which demonstrates greater automatization in postural control. The results also confirm the importance of ecological validity in investigating postural adaptations associated with sports expertise.

## Background

Sport ju-jitsu is a high-intensity, modern martial art that combines both karate and judo techniques [1]. In particular, sport ju-jitsu fights proceed in three stages: (1) Punches and kicks to the head and torso, (2) Throwing, choking, and joint locking, and (3) Floor techniques, when the competitors are kneeling, sitting, or lying on the mat. Although the floor techniques are allowed, whenever the fight is stopped by the referee, it is renewed in the standing position [1]. Therefore, one of the aims during a fight is to disturb the opponent’s balance while gripping the uniform or forcefully throwing or pushing. Maintaining a stable posture is also challenging in defensive and counterattacking techniques performed in highly unstable positions [2]. Considering the above, postural control seems to be one of the most important factors determining the effectiveness of a sport ju-jitsu athlete. However, to the best of our knowledge, there is no study investigating the relationship between postural control and expertise in sport ju-jitsu athletes or explaining the mechanisms underlying sport-specific adaptations in balance control acquired over years of training.

Thus far, it has been widely proven that training in martial arts improves postural control in patients with neurological disorders [3], the visually impaired [4], and healthy adults alike [5]. Changes in postural control were also observed as a result of long-term combat sports training in judo [6], karate [7], taekwondo [8], or wrestling athletes [9]. Generally, elite senior athletes showed superior balance measured in static and dynamic tests in comparison to untrained individuals and junior athletes [10]. On the one hand, elite judokas were characterized by a smaller radius of center of foot pressure (COP) and recovered their balance significantly faster after kicking [10]. On the other hand, increased COP displacements were found in static positions in elite adult wrestlers and karateka [9, 11]. Differences in postural stability were also observed in the early years of training, with adolescent martial arts practitioners showing a superior balance ability in comparison to their psychically active peers [12].

Many authors also emphasized that changes in postural control are more pronounced in ecological contexts, i.e. sport-specific positions or environments [13], but not in non-ecological ones, unless the task reaches the desired level of difficulty or is sufficiently specific [14]. For example, in static conditions (i.e. quiet standing), elite judokas, dancers, and surfers did not exhibit better postural stability than their intermediate peers [15–17]. However, it was possible to differentiate them while performing a dynamic postural task on an unstable surface (surfers) or in dance-like positions (dancers). It is worth mentioning that there are several studies in which the differences in postural control between martial arts athletes and healthy control subjects were observed in non-specific tasks, i.e. quiet standing [9, 11, 18] and functional tests [18].

All the above-mentioned studies assessed postural stability using force platforms, which are considered the gold standard for quantifying the measurement of COP in various populations (e.g. elite athletes). Force platforms register ground reaction forces and moments of forces, which are further used in the calculation of COP displacements. However, the complex nature of dynamic postural sway measurements that assume linearity, such as COP range, rms, or velocity, are difficult to interpret and are often insufficient for explaining the beneficial role of sports expertise on postural control. More recent findings revealed that non-linear measures of COP (e.g. rambling–trembling decomposition or sample entropy—SampEn) may improve the understanding of the physiological complexity of the postural control system [19]. The former method was proposed by Zatsiorsky and Duarte [20] and is based on the concept that the instant equilibrium point (IEP) determines the parameters of body sway and that every deviation from the body’s equilibrium (IEP trajectory) generates balance-restoring forces [21]. Therefore, the authors proposed the decomposition of COP signal into two components—rambling and trembling. While rambling “reveals the motion of a moving reference point with respect to which the body's equilibrium is instantly maintained,” the trembling component “reflects body oscillation around the reference point trajectory” [20]. Additionally, according to Zatsiorsky and Duarte the rambling trajectory may reflect the supraspinal processes which are involved in the control of IEP displacement while trembling may reflect the reflexes and mechanical properties of the muscles and joints, e.g. muscle stiffness [22]. It is also believed that trembling reflects disparities between motor planning and motor output.

SampEn, on the other hand, quantifies the regularity of a time series and is commonly used as a measure for the automatization of the postural control process [23]. Commonly, larger entropy values indicate lower regularity in the COP signal, and as a consequence higher automatization in postural control [24]. However, low values of entropy convey the high regularity of the signal and are associated with high levels of attention in the process of maintaining a stable posture.

In the present study, we would like to assess the differences in postural control between elite sport ju-jitsu practitioners and untrained control subjects in non-ecological conditions (quiet standing–QS) and ecological conditions (combat stance–CS) using the rambling-trembling and SampEn approach. This may help investigate the relative contribution of spinal and supraspinal mechanisms to postural control in expert athletes, and as a consequence improve the understanding of long-term sport ju-jitsu training adaptations. We hypothesize that there will be no differences between sport ju-jitsu athletes and control subjects during QS, however we expect to find differences in postural sway when the subjects are examined in the sport-specific CS. More specifically, elite sport ju-jitsu athletes will reduce their body sway and rambling trajectories due to their improved estimation of body dynamics in the combat position when compared to control subjects, as well as reduce their trembling trajectory, which corresponds to less noise and fewer corrections in the planned action. Finally, we hypothesize that the elite athletes will be characterized by their more automated postural control in CS, observed through higher values of sample entropy.

## Methods

### Participants

In total, twenty-one healthy male participants took part in the study (Table 1). The individuals were selected based on non-random criteria using purposive sampling. The experimental group consisted of eleven elite sport ju-jitsu athletes (inclusion criteria: male members of the Polish National Team, Europe or World Championship medalists with 7 to 12 years of training experience). The control group consisted of ten physical education students (inclusion criteria: male students who were not involved in any sport at a competitive level). The Institutional Bioethics Committee of the University of Rzeszów approved the study and all procedures conformed to the Declaration of Helsinki.

**Table 1:** Characteristics of the examined groups

	*N*	Sex	Age (mean ± SD) [years]	Height (mean ± SD) [cm]	Weight (mean ± SD) [kg]
Ju-jitsu	11	M	17 ± 1.5	178 ± 5.2	62 ± 19.6
Control	10	M	22 ± 1	179 ± 4.9	80 ± 10.4

### Study design

To assess postural stability, we used a force plate (AMTI, AccuGait, Watertown, MA, USA) by which the ground reaction forces and moments of forces were registered at a 100 Hz sampling frequency.

**Fig. 1:**
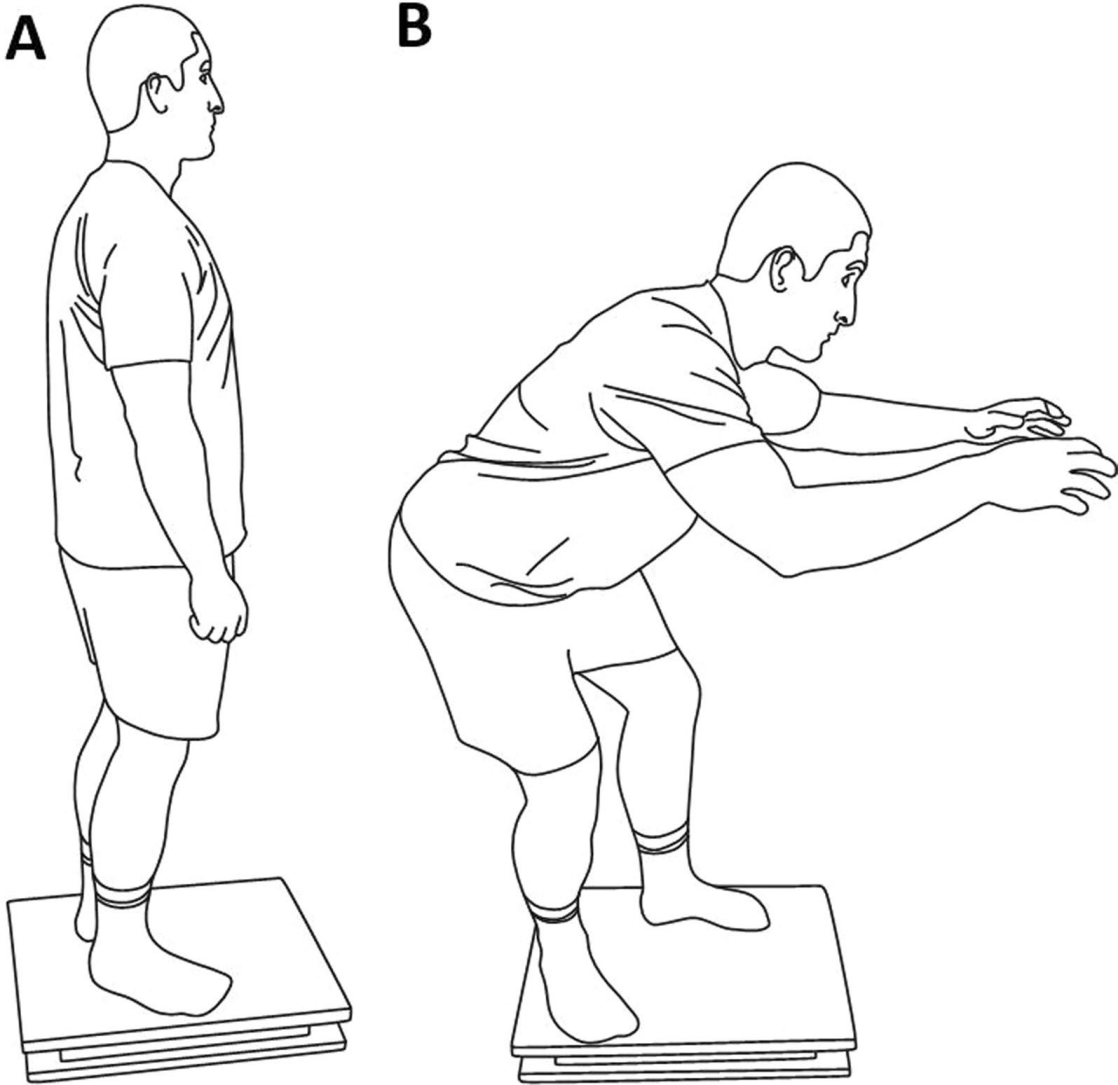
Two experimental conditions: **A** quiet standing and **B** ju-jitsu combat stance

**Fig. 2:**
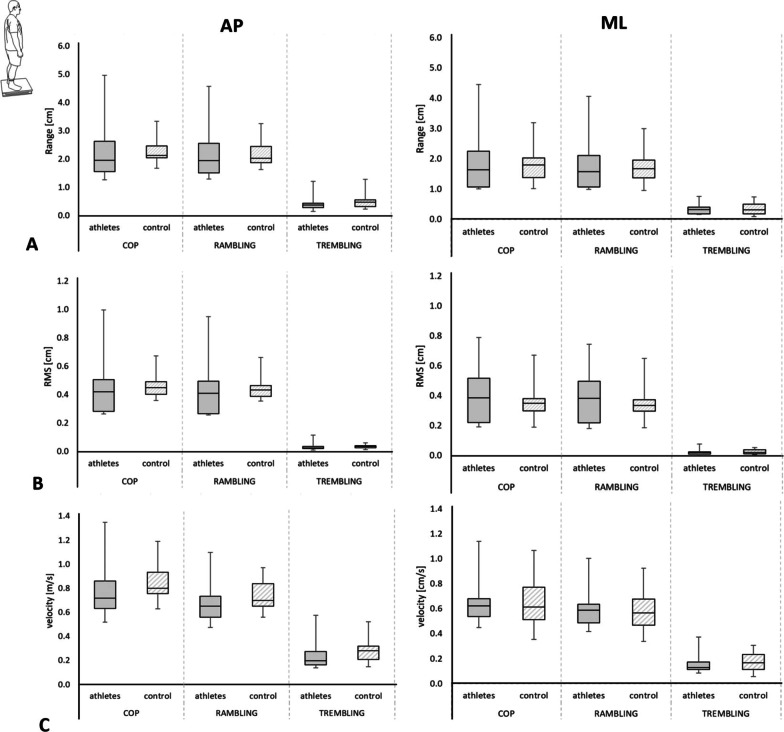
Comparison between groups in quiet standing. *Legend* **A** range of COP, rambling, and trembling, **B** root mean square (RMS), rambling, and trembling, **C** COP velocity, rambling and trembling component in the anterior–posterior (AP) and mediolateral (ML) directions (median, upper, and lower quartile, min. and max. marked as error bars) (*p < 0.05)

The experimental procedure included measurements in two types of stances: (1) QS, during which the participants were standing barefoot on the force plate with feet comfortably aligned and arms along the torso (Fig. 1A), and (2) CS which was characteristic for the initial ju-jitsu stance during combat (Fig. 1B). In CS, the participants stood with their feet shoulder-width apart with one foot in front. The arms were extended in the direction of the “opponent” with the hands situated at chest level. The hips were bent, thus the torso and thighs were at an angle of approximately 90 degrees. As a result, the torso was strongly tilted forward. In CS, all of the participants were instructed to distribute their bodyweight equally among both legs. Before the measurement started, both groups were familiarized with the CS requirements and could perform as many habituation trials as required. The measurements started after the participant declared their readiness.

In both QS and CS trials, the participants were instructed to maintain a stable posture (minimalize body sway as much as possible) with their gaze fixated at a reference point located 3 m away in front of them at eye level. The QS and CS trials were repeated 3 times and lasted for 30 s each.

### Data processing

The raw data from the force plate was processed offline using Matlab r2020b software (Mathworks Inc., Natick, MA, USA) with a 7 Hz, fourth-order, low-pass Butterworth filter. The mean value of the 3 trials were calculated for each variable. The following 3 subsets of posturographic variables in the anterior–posterior (AP) and mediolateral (ML) directions were extracted:

1. *Spatio-temporal measures of COP:* (a) Range of COP (raCOP) [cm]–maximum excursion of the COP in a given direction, (b) Velocity of COP (vCOP) [cm/s] – ratio of the total length of the COP trajectory and the recording time length, (c) Root mean square of COP (rmsCOP) [cm]–the displacements of the COP around the mean COP.
2. *Rambling-trembling decomposition of COP:* Zatsiorsky and Duarte’s [20] method for COP decomposition was used to obtain the rambling (raRAMB, vRAMB, rmsRAMB) and trembling (raTREMB, vTREMB, rmsTREMB) components of the variables listed above.
3. *Sample entropy measures of COP:* SampEn is the negative logarithm of the probability that a data set of length N, having repeated itself for m samples within a tolerance r, will also repeat itself for m + 1 samples, without allowing self-matches. The parameters m and r must be fixed for each calculation; m is the length of sequences to be compared, and r is the tolerance for accepting matches. To select the input parameters of the algorithm we used the criterion proposed by Richman and Moorman [25] and Lake et al. [26]: m = 3 and r = 0.2 × the standard deviation of the data set.

### Statistics

The Shapiro–Wilk test was used to check the data for a normal distribution, while variance homogeneity was investigated with Levene’s test. In order to compare the posturographic variables between the groups independently in QS and CS, the non-parametric Mann–Whitney U test was used. The level of statistical significance was adopted for the value of *p* < 0.05. The effect size was calculated according to the formula r = Z/$\surd$ N and reported as the r value. Statistical analyses were performed using Statistica v.13.3 (TIBCO Software Inc.).

## Results

### Comparison of elite ju-jitsu athletes and control subjects in QS

**Fig. 3:**
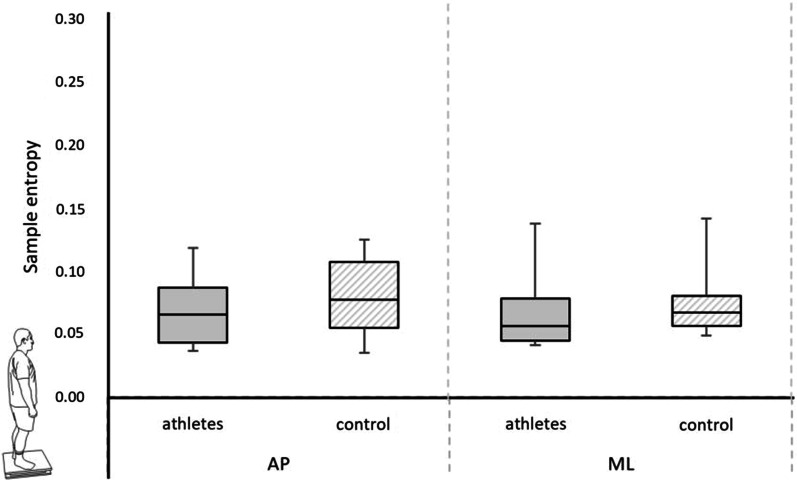
Comparison between groups of the sample entropy in quiet standing. *Legend* anterior–posterior (AP) and mediolateral (ML) directions (median, upper, and lower quartile, min. and max. marked as error bars) (*p < 0.05)

No differences between groups were observed in the spatio-temporal parameters of COP, rambling-trembling, and sample entropy in QS (*p* > 0.05) (Figs. 2 and 3).

### Comparison of elite ju-jitsu athletes and control subjects in CS

**Fig. 4:**
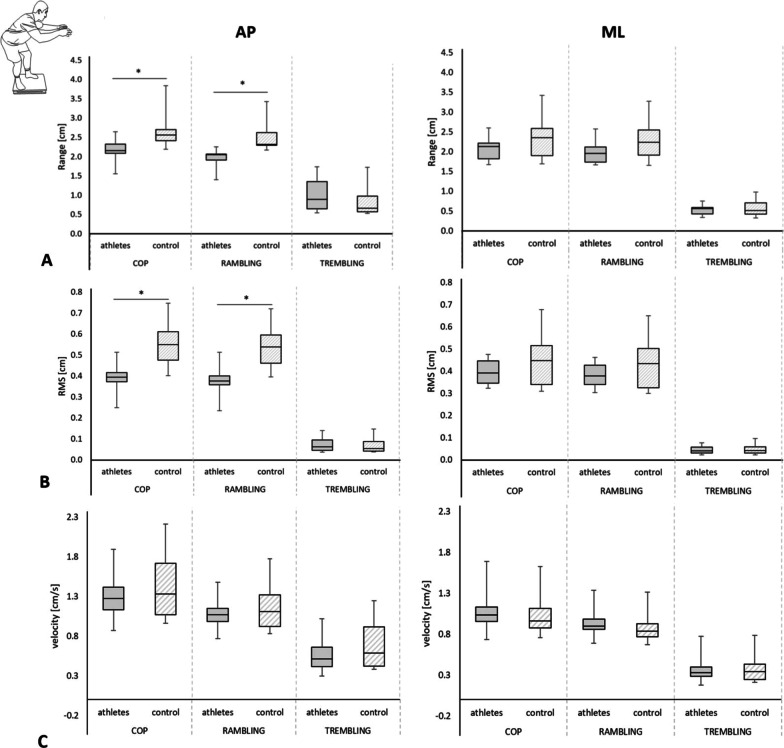
Comparison between groups in combat stance. *Legend* **A** range of COP, rambling, and trembling, and **B** root mean square (RMS), rambling, and trembling, **C** COP velocity, rambling and trembling component in the anterior–posterior (AP) and mediolateral (ML) directions (median, upper, and lower quartile, min. and max. marked as error bars) (*p < 0.05)

Elite ju-jitsu athletes showed a smaller body sway than control subjects in AP, which were observed in the spatio-temporal parameters of COP: raCOP (U = 13, *n*_1_ = 11, *n*_2_ = 10, *p* < 0.01, r = 0.64) and rmsCOP (U = 9, *n*_1_ = 11, *n*_2_ = 10, *p* < 0.01, r = 0.70) (Fig. 4A, B), as well as in the rambling parameters: raRAMB (U = 2, *n*_1_ = 11, *n*_2_ = 10, *p* < 0.01, r = 0.81) and rmsRAMB (U = 9, *n*_1_ = 11, *n*_2_ = 10, *p* < 0.01, r = 0.70) (Fig. 4A, B). No differences between groups were found in the AP (vCOP, vRAMB, and vTREMB) and ML direction (all variables) (*p* > 0.05) (Fig. 4C).

**Fig. 5:**
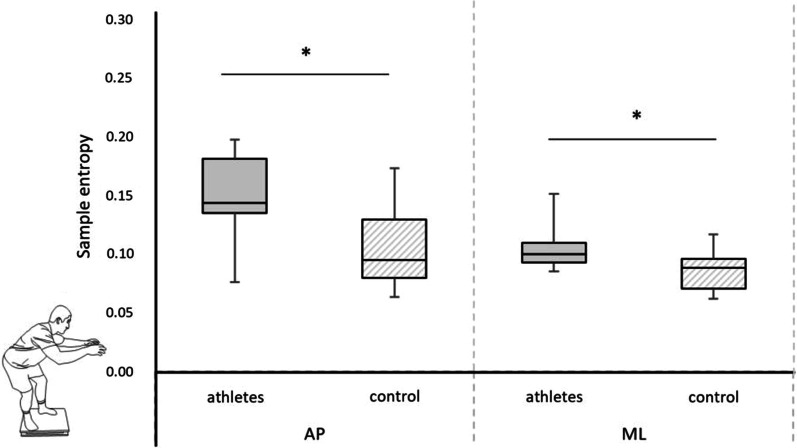
Comparison between groups of the sample entropy in combat stance. *Legend* anterior–posterior (AP) and mediolateral (ML) directions (median, upper, and lower quartile, min. and max. marked as error bars) (*p < 0.05)

The sport ju-jitsu athletes presented higher values of SampEn in CS in the AP direction (U = 17, *n*_1_ = 11, *n*_2_ = 10, *p* < 0.01, r = 0.58) and ML (U = 26, *n*_1_ = 11, *n*_2_ = 10, *p* = 0.04, r = 0.44) (Fig. 5).

## Discussion

The aim of the present study was to assess the differences in postural control between elite sport ju-jitsu athletes and untrained control subjects in QS and CS and explain the mechanisms underlying the benefits of sport ju-jitsu expertise on postural control. We hypothesized that differences in postural control will be only observed in sport-specific CS; in particular, elite sport ju-jitsu athletes will reduce their body sway and rambling trajectories in CS due to improved estimation of body dynamics, as well as reduce their trembling trajectory, which corresponds to less noise and fewer corrections in the planned action. Additionally, we hypothesized that the elite athletes will be characterized by more automated postural control in CS, observed in higher values of sample entropy.

The results confirmed the first hypothesis that no difference will be found in postural control in QS between elite sport ju-jitsu athletes and control subjects. These results are in line with previous findings investigating the effect of expertise on the non-ecological context of postural control (static balance) among judokas, surfers, and dancers [15–17, 27]. Moreover, differences between elite sport ju-jitsu athletes and controls were observed in CS (ecological conditions). This supports the idea that the nature of long-term sports practice leads to the acquisition of specific postural regulations presented in sport-specific positions and environments. Paillard [28] compared the postural stability of judokas who practice their favorite throwing technique in a monopedal and bipedal stance; the results showed that in the assessment of double leg support, judokas who practice throwing in a bipedal stance were more stable than those who trained in a monopedal stance, and conversely, in the assessment of single leg support, superior balance was found in judokas who train in single leg support. Similar results have been observed by Casabona et al. [27], who analyzed COP in stances with different foot configurations in ballet dancers. Differences between the experts and control group were only found in the stance in which the foot configuration matched the needs of ballet practice.

The superior postural performance in CS by sport ju-jitsu athletes was characterized as the ability to minimize postural sway. This was observed in lower values of COP range and rms. Many previous findings associated smaller postural sway with better postural skills [17, 27, 29–32]. For example, ballet dancers exhibited lower values of COP area, range, and rms than non-dancers in a feet configuration representing a ballet-specific position [27]. Similarly, significant differences between elite dancers and control subjects were found in dance-like techniques, which required weight transfer on the ball of the foot, on an unstable surface, with and without vision, and in ballet jumps, followed by maintaining the new, stable posture [17]. In the study by Gauchard et al. [33], reduced body sway was observed in karatekas when compared to control subjects, especially in more challenging conditions, i.e. standing with eyes closed on a foam surface.

The greater precision of postural control in elite ju-jitsu athletes may correspond to the contribution of sensory-motor pathways (proprioceptive, vestibular and visual) in balance control during CS. It is generally accepted that the repetitive practice of motor tasks in a sport-specific environment results in neurological adaptations which involve the increased exploitation of some sensory inputs at the expense of the others [14]. For example, athletes who train on an unstable surface, i.e. a soft mat such as wrestlers, were found to be proprioception-dependent, while boxers who compete on a stable surface relayed visual information to a greater extent [34]. Similarly, judokas exhibited a better postural performance than ballet dancers in conditions with eyes closed in comparison to eyes opened [6]. In fact, judokas train in a constantly changing environment due to their opponents’ actions, therefore giving greater importance to proprioceptive and vestibular information [6]. However, to confirm how the discussed sensory pathways influence postural regulation in elite ju-jitsu athletes, the methodological approach should include attempts with eyes closed. The lack of this data can be considered as one of the limitations of the present study.

The control group was characterized by increased COP range and rms in comparison to expert athletes, which may reflect the exploratory behavior of postural sway [35]. According to Carpenter et al. [36], increased sway is a part of the perception–action strategy that allows the position of the body to be tracked relative to the limits of stability and enhances the quality and volume of sensory information. In particular, a greater variety of sensory receptors can be stimulated in dynamic conditions and allows the better integration of reliable afferent information. Based on the exploratory hypothesis, it is possible that the central nervous system “helps” non-athletes to maintain balance in CS by purposefully increasing COP displacement to ensure the sufficient quality of sensory information.

Enhancing somatosensory information was also introduced as an explanation of increased body sway in elite athletes, such as wrestlers or karatekas [9, 11, 18], biathletes, runners, or cyclists [13] when compared to controls. The authors mentioned above proposed that sports expertise leads to redundancy in the sensorimotor system, which is based on specific adaptations at the spinal and supraspinal level. However, standard spatio-temporal parameters of COP are not always sufficient for addressing the problem of explaining the complex nature of postural sway. Therefore, they should be supported by the analysis of non-linear COP measures, i.e. rambling-trembling and SampEn.

Zatsiorsky and Duarte [20] proposed that rambling reflects the migration of the reference point about which the equilibrium is maintained and represents the supraspinal contribution to sway. Trembling, on the other hand, reflects the oscillations around the reference point and represents the contribution from spinal reflexes and a change in the mechanical properties of the muscles. We observed that sport ju-jitsu athletes were characterized by a decreased rambling component of COP range and rms when compared to non-athletes. These results support our hypothesis that the decreased sway in the examined athletes results from the smaller migration of IEP. On the other hand, the larger sway in CS in the control group is due to greater IEP migration. The proportion of the rambling and trembling components in the overall sway was assessed by Ferronato and Barela [37] when describing age-related changes in postural control. The authors showed that the increased rambling component of sway in children is due to difficulties in estimating the COP position when compared to adults. The authors believed that with age, the central nervous system receives more accurate and reliable information regarding body dynamics and improves the estimation of overall body position. This belief is called into question if the same mechanism underlies the reduced sway in ju-jitsu athletes; however, the results of increased rambling in the control group are logical from an exploratory theory standpoint, which explains increased body sway as a strategy for enhancing somatosensory resources.

In contrast to our expectations, there was no difference between athletes and controls in the trembling component, suggesting that the difference did not include the reflexive corrections of COP trajectory at the spinal level. According to Zatsiorsky and Duarte [21], the lack of differences in the examined groups might also be a sign of consistency between the planned action and the action that was actually performed.

As expected, no significant differences between groups were found in QS, while in CS the elite ju-jitsu athletes were characterized by significantly higher values of sample entropy when compared to the control subjects. According to Borg and Laxaback [23], the high entropy and chaotic excursion of COP can be interpreted as a sign of a healthy, vigilant system, but also as an effective strategy for maintaining body balance. What is more, according to Donker et al. [38], sway regularity was also found to be positively correlated with the amount of attention invested in postural control. In another words, the more irregular the signal is, the less attention is devoted to balance control. Based on our results, it appears that when elite ju-jitsu athletes set the initial CS, posture control is mainly handled on “auto-pilot” so that a greater amount of attention is allocated to observe and analyze the behavior of the opponent. This explanation is in strong agreement with the recent study by Rhea et al. [39], which emphasized that the external focus of attention can be used to increase postural control entropy in young and older adults.

The automatization of postural control in elite athletes was discussed in the study by Kuczyński et al. [40] investigating the effect of dual tasking on postural control in dancers. According to the authors, elite dancers were able to act at the same level of effectiveness in dual tasking (postural task combined with mental task), while non-athletes could not devote the desired level of attention to postural control, and as a consequence presented worse stability. It has been proposed that the automatization of postural control in dancers was associated with the environment in which they perform. Dancers must invest much attention in fast decision-making concerning their body position and direction of movement in space, and in many situations anticipate the position of their partners. If that is the case, it is not surprising that martial arts athletes present more automated postural control than their non-trained peers.

To the best of our knowledge, this is the first study which examines postural control in elite ju-jitsu athletes. This is not a trivial act as various kinds of ju-jitsu are some of the fastest-growing martial arts in the world. At the same time, ju-jitsu is one of the few martial arts which is not sufficiently described in the literature in terms of postural control. Apart from the strengths of the present study, a number of potential limitations should be considered. The first limitation, as discussed earlier, was not including trials with eyes closed. Therefore, postural strategies corresponding to the preferential involvement of sensory-motor pathways could not be addressed. The second limitation was the age differences between the athletes and control subjects. Although all ju-jitsu athletes included in the study were post-PHV (peak height velocity), the research should be repeated for a group of subjects with similar ages. Third, the sample size, although representative, was too small to generalize the results for a larger population. A study with a larger sample size would be of benefit to support the presented evidence.

## Conclusion

The present study provided evidence that elite sport ju-jitsu athletes and control subjects had comparable postural control in QS. The sport-specific adaptations were observed in ju-jitsu CS and revealed that the examined athletes had smaller postural sway and its rambling component compared to non-athletes. This might indicate the more precise control of COP and improved ability in estimating the COP position in the examined group of athletes. The SampEn results confirmed that the COP pattern of sport ju-jitsu athletes was more irregular, which demonstrates greater automation in postural control. Our results also confirm the importance of ecological validity in investigating postural adaptations associated with sports expertise.

## Data Availability

The data is available from the corresponding authors upon request.

## Abbreviations

AP: Anterior–posterior
ML: Mediolateral
QS: Quiet standing
CS: Combat stance
COP: Center of foot pressure
raCOP: Maximum excursion of the COP in a given direction
vCOP: Ratio of the total length of the COP trajectory and the recording time length
rmsCOP: Displacements of the COP around the mean COP
raRAMB, vRAMB, rmsRAMB: Rambling of spatio-temporal measures of COP
raTREMB, vTREMB, rmsTREMB: Trembling of spatio-temporal measures of COP
SampEn: Sample entropy
